# *In vivo* imaging approach to detect and quantify early fibrogenic remodeling following low-dose bleomycin exposure

**DOI:** 10.3389/fphar.2026.1792856

**Published:** 2026-04-09

**Authors:** Elena Mantovani, Beatrice Ragnoli, Nausicaa Clemente, Fausto Chiazza, Mario Malerba

**Affiliations:** 1 Department of Pharmaceutical Sciences, Università del Piemonte Orientale “Amedeo Avogadro”, Novara, Italy; 2 Department of Traslational Medicine, Università del Piemonte Orientale “Amedeo Avogadro”, Novara, Italy; 3 Pulmonology Department, Ospedale S. Andrea, Vercelli, Italy; 4 Center IPAZIA, Novara, Italy

**Keywords:** early interstitial lung diseases, imaging, inflammation, micro-CT, preclinical

## Abstract

**Introduction:**

Interstitial lung diseases (ILDs) are a heterogeneous group of pulmonary disorders characterized by variable inflammation and fibrosis, leading to progressive impairment of gas exchange. Current therapeutic strategies rely on immunosuppressants and antifibrotics, but early diagnosis and treatment remain crucial to improve long-term outcomes. The development of targeted therapies requires accurate preclinical models that reproduce the initial disease stages.

**Methods:**

Here, we propose an *in vivo* model based on chronic administration of low-dose subcutaneous bleomycin (BLM, 30 U/kg) to induce early lung injury. To detect early fibrotic alterations, we implemented a micro-CT segmentation strategy, dividing the lung parenchyma into ten aeration-based regions.

**Results:**

This approach enabled the identification of subtle changes in aeration on day 14 after BLM exposure. Further molecular characterization by Western blot analysis revealed, at day 21, significant activation of pro-inflammatory and pro-fibrotic pathways, including upregulation of the TGF-β/Smad 2-3 signaling cascade and induction of the NLRP3 inflammasome pathway. These findings confirm that low-dose BLM is sufficient to initiate pathogenetic mechanisms relevant to ILD.

**Discussion:**

Overall, our study establishes a novel preclinical protocol integrating reduced BLM dosage with refined micro-CT analysis, providing a promising platform for investigating early ILD pathogenesis and facilitating translational research aimed at optimizing therapeutic intervention windows.

## Introduction

Interstitial lung disease (ILD) comprises a heterogeneous group of pulmonary disorders characterized by varying degrees of inflammation and fibrosis within the lung interstitium, leading to impaired gas exchange and progressive respiratory failure (Althobiani et al., 2024).

Since there are substantial variations in prognosis and treatment among the different types and severities of ILD, an early and accurate diagnosis as a timely intervention may improve long-term outcomes and potentially alter disease trajectories (Mori et al., 2015).

Technological advances in lung imaging led to the description of Interstitial lung abnormalities (ILAs), incidental radiological findings suggesting fibrotic abnormalities thought to represent early or mild pulmonary stage of fibrosis (Hatabu et al., 2020). ILAs have been associated to an increased risk of progression to clinically significant ILD and death (Putman et al., 2016), highlighting the need for early detection and intervention (Spagnolo et al., 2021).

Current therapeutic strategies for ILD primarily involve orally administered immunosuppressors and antifibrotic agents such as pirfenidone and nintedanib. While these treatments have demonstrated efficacy in slowing disease progression, they are often associated with substantial side effects, including gastrointestinal disturbances and hepatotoxicity, which can limit patient adherence and overall treatment effectiveness (Richeldi et al., 2014; Fournier et al., 2022).

To develop more effective and targeted therapies, robust preclinical models that accurately recapitulate human disease are essential. The bleomycin-induced lung injury model is the most established animal model for studying pulmonary fibrosis due to its reproducibility and the histopathological similarities it shares with human ILD (Moeller et al., 2008). The administration of bleomycin causes a dose-dependent lung inflammatory response, with activation of cytokines such as transforming growth factor-β (TGF-β) and connective tissue growth factor (CTGF) that contribute to the development of the repair phase and fibrosis (Della Latta et al., 2015).

This observation led to the assumption that bleomycin reproduces typical features of the human disease and hence, the use of this model has become very popular. Traditionally, this model involves the administration of a single high dose of bleomycin, leading to acute lung injury followed by fibrotic changes (Fournier et al., 2022; Buccardi et al., 2023). However, such models predominantly represent advanced stages of fibrosis and may not adequately mimic the early inflammatory events that precede fibrogenesis in human ILD (Della Latta et al., 2015).

Recent studies have highlighted the importance of dosing and timing in the bleomycin model to better simulate the disease’s progression. For instance, systemic administration of bleomycin at lower doses over extended periods has been shown to induce a more gradual onset of fibrosis, closely resembling the chronic nature of human ILD (as reviewed in Della Latta et al. (2015)). Additionally, imaging modalities like MRI, PET and micro-CT have been employed to monitor disease progression in these models, providing insights into the temporal dynamics of lung injury and repair (Moeller et al., 2008).

Besides, the route of bleomycin administration significantly influences the pattern and severity of lung injury. Intratracheal instillation often results in localized injury, whereas systemic routes, such as intravenous or subcutaneous administration, can lead to diffuse pulmonary involvement, better reflecting the heterogeneity observed in human ILD (Della Latta et al., 2015; Degryse and Lawson, 2011). Adjusting the administration route and dosing regimen can, therefore, enhance the translational relevance of the model, but with the current analysis methods, which usually segment lung parenchyma only between well and poorly aerated tissue, it is impossible to discern the earliest stages of the disease, meaning those most relevant for a successful pharmacological intervention.

In summary, while the bleomycin-induced lung injury model has been instrumental in advancing our understanding of pulmonary fibrosis, modifications to the dosing strategy, administration route and *in vivo* imaging analysis are essential to develop preclinical models that accurately represent the early stages of ILD.

In this paper we propose an *in vivo* preclinical model to study the earliest phases of ILD which involves the chronic administration of low doses of bleomycin s.c. and take advantage of a refined way to finely dissect and segment lung parenchyma.

## Materials and methods

### Animals and micro-CT imaging

All animal procedures were conducted in accordance with Italian regulations (D.L.26/2014) and European Directive 2010/63/EU, with approval from the Organismo Preposto al Benessere Animale (OPBA) of the Università del Piemonte Orientale, Novara, Italy (approval no. DB064.85). Female C57BL/6J mice aged 6–7 weeks (n = 21) were weighed and randomly assigned to receive either bleomycin (BLM n = 11 BLM+ n = 1) or sham treatment (n = 9).

**FIGURE 1:**
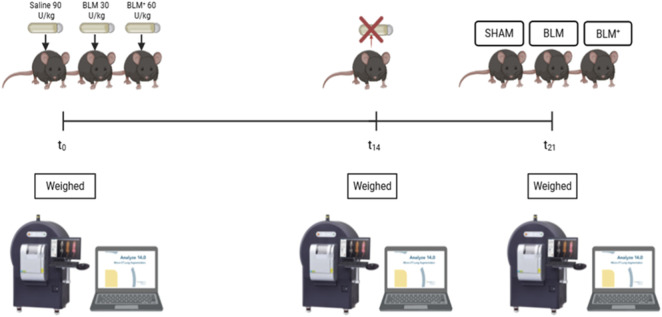
Experimental design. At different time points, twenty-one female C57BL/6J mice were weighed and subjected to micro-CT scanning. From day 0 to day 14, pulmonary fibrosis was induced using bleomycin-filled osmotic minipumps. At the experimental endpoint (day 21), fibrosis induction was assessed by micro-CT and *ex vivo* analyses.

At baseline (day 0), mice were weighted and underwent high-resolution micro-CT imaging (QuantumGX, PerkinElmer®, Inc.) with 72 μm voxel size using the following parameters: 90 kV, 88 μA, 36 mm field of view for 4 min. Concurrently, osmotic minipumps (ALZET 1007D, DURECT Corporation, Cupertino, CA, USA; release rate 0.5 μL/h) containing either bleomycin (30 U/kg, BLM group; 60 U/kg, BLM+ group; Bleoprim, Sanofi S.r.l.) or saline (0.9% NaCl, Sham group) were implanted subcutaneously in the intrascapular region. On day 14, minipumps were explanted, mice were weighed, and micro-CT imaging was repeated as detailed above. On day 21, mice were weighed and underwent final micro-CT imaging before euthanasia by intracardiac exsanguination under anesthesia. Micro-CT acquisitions were performed without respiratory gating. All animals were scanned under identical anesthetic and acquisition conditions to ensure internal consistency across time points. The experimental *in vivo* protocol is represented in Figure 1. Blood samples were collected and plasma separated by centrifugation 1,400 *g* for 10 min. Right lung specimens were immediately fixed in 4% paraformaldehyde and processed for paraffin embedding according to standard protocols, while left lungs were snap frozen for molecular analysis.

All procedures were performed under anesthesia with 3.5% isoflurane in oxygen for induction, maintained at 2.5% isoflurane.

### Image segmentation and analysis

Micro-CT images were analyzed using Analyze 14 software (AnalyzeDirect, Inc.). Semi-automated segmentation was performed on lungs object extracted from two-dimensional images based on Hounsfield Units (HU). Lung parenchyma segmentation was performed using a range between −900 HU and −100 HU following system calibration and normalization according to manufacturer specifications. This interval corresponds to lung tissue as described in CT densitometry literature, where air approximates −1,000 HU and water 0 HU (Ravanetti et al., 2021). Identical threshold settings were applied across all animals and time points to ensure consistency. According to literature, the total volume of lungs parenchyma has been divided into two main HU-based regions, to represent its well-aerated (−900/−501) or poorly-aerated (−500/−100) regions (Ravanetti et al., 2021). Thereafter we tried to improve this analysis by dissecting lung parenchyma not into two but into 10 equal segmentations based on HU. The HU interval between −900 HU and −100 HU (800 HU total range) was subdivided into ten equal, non-overlapping bins of 80 HU each (−900/−821; −820/−741; −740/−661; −660/−581; −580/−501; −500/−421; −420/−341; −340/−261; −260/−181; −180/−100 HU). These predefined thresholds were applied identically across all animals and time points to ensure reproducibility.

For both methods, resulting segments volumes (mm^3^) were expressed as percentages on total lung volume.

### Histological analysis

Whole lung sections (5 μm thickness) were prepared using a Leica RM2125 RTS microtome. Following deparaffinization and rehydration, slides were stained with Masson’s trichrome to visualize collagen deposition and tissue architecture. Briefly, sections underwent overnight fixation in Bouin’s solution (Sigma Aldrich HT10132), followed by washing in running tap water. Sections were then stained with iron hematoxylin (Sigma Aldrich HT107) for 5 min, washed in running tap water for 5 min, and rinsed in deionized water. Subsequently, slides were stained with Biebrich scarlet acid fuchsin (Sigma Aldrich HT151) solution for 5 min and rinsed in deionized water. Sections were then treated with working Phosphotungstic acid solution (Sigma Aldrich P4006) for 5 min, followed by aniline blue solution (Sigma Aldrich B8563) for 5 min. After treatment with 1% acetic acid for 2 min, slides were dehydrated through graded alcohols to xylene and mounted with coverslips. Digital microscopy was performed using a ZEISS Axioscan 7 whole-slide imaging system with ZEN Lite 3.5 software under brightfield illumination at 40× magnification using the REF_BF H&E Slide acquisition protocol.

### Western blot analysis

Nuclear and cytosolic protein extracts were prepared as previously described (Pinton et al., 2024). Protein concentrations were determined using Bradford assay (B6916 Sigma-Aldrich) following manufacturer instructions, and extracts were stored at −80 °C until use. Western blot analyses were performed on random animals from the same experimental cohort used for micro-CT imaging. Depending on protein availability for each specific target, 4–7 independent biological replicates per group were analyzed. For each sample, 15 μg of nuclear or 50 μg of cytosolic proteins were loaded onto SDS-PAGE gels for electrophoretic separation and transferred to polyvinylidene difluoride (PVDF) membranes. Non-specific binding sites were blocked with milk proteins (5% in TBS) for 2 h and then incubated overnight with the following primary antibodies (in 5% milk in TBS-Tween): rabbit anti-SMAD2/3 (Cell Signalling #8685, 1:1,000 dilution), mouse anti-caspase-1 (p10) (Adipogen AG-20B-0044, 1:1,000 dilution), mouse anti-NLRP3 inflammasome (Adipogen AG-20B-0014, 1:1,000 dilution), rabbit anti-TGF-β1 (Abcam ab215715, 1:1,000 dilution), and mouse anti-IL-1β (Cell Signaling #12242, 1:1,000 dilution). Secondary antibodies were: HRP-conjugated anti-mouse (Cell Signaling #7076, 1:2,000 dilution) and anti-rabbit (Cell Signaling #7074, 1:2,000 dilution). Membranes were developed using enhanced chemiluminescence (ECL) and imaged with a Bio-Rad ChemiDoc™ imaging system. Results were normalized to β-actin expression (mouse anti-β-actin, Sigma Aldrich A5441, 1:5,000 dilution) and to Sham group.

### Statistical analysis

**FIGURE 2:**
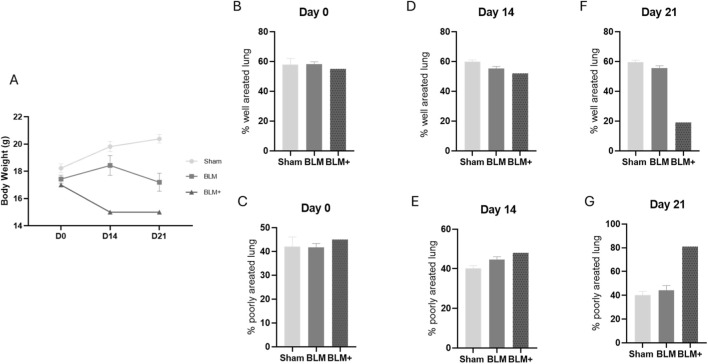
**(A)** mice body weight at the different time points (Sham; BLM; BLM+). **(B–G)** standard quantitative method for evaluating lung parenchymal aeration status in mice, expressing the percentage of well-aerated and poorly-aerated tissue at various time points. Data indicates that this approach identifies advanced tissue densification but lacks sensitivity for early alterations. Statistical comparisons were performed between Sham and BLM 30 U/kg groups. The BLM+ group (n = 1) is shown for descriptive purposes only and was not included in statistical testing.

**FIGURE 3:**
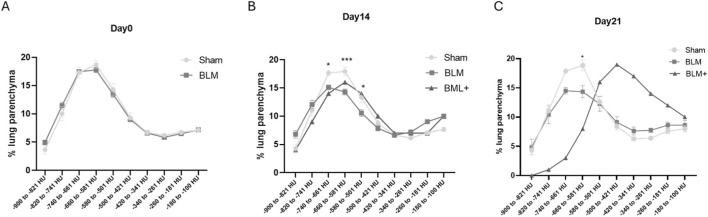
Refined segmentation strategy. Novel method for measuring respiratory parenchymal aeration status in mice. Sham; BLM; BLM+. **(A)** Day 0. **(B)** Day 14. **(C)** Day 21. Day 14: Reduction of well-aerated compartments in BLM-treated mice, consistent with early parenchymal densification. Day 21: Persistent aeration shift in BLM 30 U/kg and marked rightward shift in BLM+, reflecting advanced loss of air fraction. *p < 0.05 vs. Sham; **p < 0.01 vs. Sham; ***p < 0.001 vs. Sham. Statistical comparisons were performed between Sham and BLM 30 U/kg groups. The BLM+ group (n = 1) is shown for descriptive purposes only and was not included in statistical testing.

Statistical analyses have been conducted with GraphPad Prism 8.0.2. For aeration analysis in Figure 2, comparison was performed between Sham and BLM 30 U/kg groups using an unpaired two-tailed Student’s t-test. When variance heterogeneity was detected, Welch’s correction was applied. For aeration analysis in Figure 3, two-way ANOVA was performed separately at each time point, with treatment group (Sham vs. BLM 30 U/kg) and HU bin as factors, followed by Dunnett’s multiple comparisons test. Analyses were conducted separately for each time point. The BLM+ (60 U/kg) condition consisted of a single animal and was included solely as a descriptive reference control. This group was not included in any inferential statistical analyses.

Western blot data (Sham vs. BLM 30 U/kg) were analyzed using an unpaired two-tailed Student’s t-test. Each data point represents an independent biological replicate. Welch’s correction was applied when appropriate. P values < 0.05 were considered as statistically significant. Data are indicated as mean ± SEM.

## Results

### Early ILD is not detectable with conventional micro-CT images analysis

On day 0 all mice from the different groups showed a comparable body weight. BLM administration led to a decrease in mice body weight that was detectable on day 14 (BLM+) and on day 21 (BLM group) if compared to sham treated mice (Figure 2A).

On day 0, day 14 and day 21, lung parenchyma was digitally dissected in well and poorly aerated areas using analysis protocol described in literature (Ravanetti et al., 2021). As expected, on day 0 no differences were observed between the groups (Figures 2B,C), and on day 14 only a not significant trend of reduction and increase in respectively well (Figure 2D) and poorly (Figure 2E) aerated parenchyma was observed in BLM and BLM+ groups if compared to sham. On day 21, a visible alteration in lung aeration was observed only in the mouse treated with high doses of BLM (BLM+, 60 U/kg), while no differences could be detected in the group treated with lower doses of BLM if compared to sham group (Figures 2F,G).

### Early ILD can be detected with a new approach of micro-CT images analysis

As early ILD was not detectable with previous approaches, we implemented a new protocol for digital lung parenchyma segmentation consisting in dissecting lung parenchyma into 10 different segments from the most to the less aerated. Data obtained were expressed in percentage of lung tissue in that segment on total lung volume.

With this setup, no differences were observed at baseline (day 0) between the different groups (Figure 3A). Conversely, on day 14 we observed an alteration in lung aeration in both groups receiving low and high dose of BLM consisting in a significant reduction of the values on the left segment of the curve (representing well aerated parenchyma) if compared to sham treated mice (Figure 3B). On day 21, BLM mice maintained the impairment in lung aeration observed on day 14. Besides, the administration of 60 U/kg of BLM (BLM+), led to a complete shift of the pick of the curve to the right side of the graph, indicating a strong impairment in lung function (Figure 3C).

**FIGURE 4:**
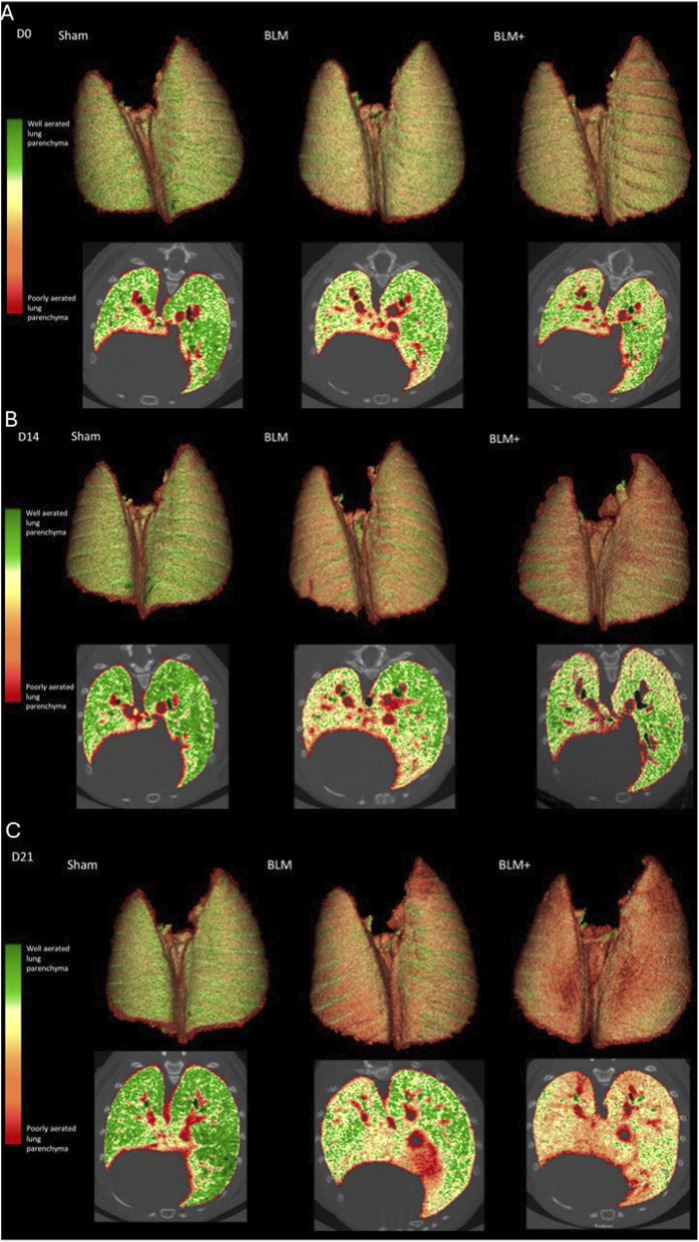
Segmented representative images. Three-dimensional and axial images of segmented mouse lungs. From left to right: Sham, BLM, BLM+ at various time points. **(A)** Day 0. **(B)** Day 14. **(C)** Day 21. Color coding reflects the predefined HU-based aeration bins, with a gradient ranging from highly aerated regions (green, lower-density HU values) to poorly aerated regions (red, higher-density HU values). Early reduction of highly aerated regions is evident at day 14, with pronounced densification in BLM treated groups at day 21.

A representation of the segmentation of lung parenchyma at the three time-points of the three groups can be observed in Figure 4.

**FIGURE 5:**
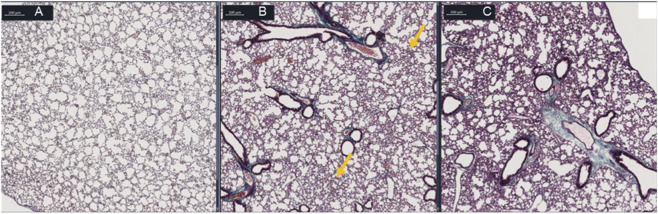
Histological analysis. Representative images of Masson’s trichrome stained mice lung slides. **(A)** Sham. **(B)** BLM. **(C)** BLM+. BLM 30 U/kg shows mild inflammatory thickening without overt collagen deposition (early remodeling), whereas BLM+ displays more pronounced architectural alteration.

On day 21, lungs from different groups were collected to evaluate their morphology. Here we documented, in affected areas, a mild inflammation composed predominantly of mononuclear cells and a gradient in the tissue thickening in bronchoalveolar walls and lung parenchyma between sham, BLM and BLM+ mice (Figure 5). Finally, on day 21, no collagen deposits were observed in BLM group (Figure 5), a condition that can properly represent the early phases of ILD, a time window in which pharmacological intervention is still exploitable.

### Profibrotic pathways are affected in the lungs of mice with early ILD onset

To further characterize our preclinical model, we evaluated the expression and activation of the TGF-β dependent signaling pathway, known to be strongly involved in the early phases of ILD (Thomas et al., 2016) in Sham and BLM 30 U/kg treated groups.

**FIGURE 6:**
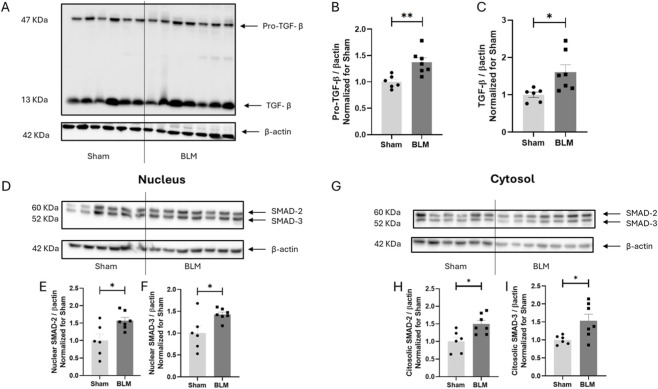
TGF-β/Smad pathway activation in BLM mice. **(A)** representative Western blot analysis of cytosolic TGF-β mature (13 kDa) and precursor (47 kDa) forms in BLM and Sham mice lungs, normalized to β-actin. **(B,C)** Pro-TGF-β and TGF-β expression normalized to β-actin and sham controls. Panel D and G: representative Western blot analysis of nuclear **(D)** and cytosolic **(G)** SMAD2/3 in BLM and Sham mice lungs, normalized to β-actin. Panel E and F nuclear expression of SMAD2 **(E)** and SMAD3 **(F)** expression normalized to β-actin and sham controls. Panel H and I: cytosolic expression of SMAD2 **(H)** and SMAD3 **(I)** normalized to β-actin and sham controls.

The administration of 30 U/kg of BLM led to a statistically significant increase in both the precursor (Figures 6A,B) and the active (Figures 6A,C) forms of TGF-β, indicating respectively a higher expression and activation of this protein.

TGF-β leads to the activation and translocation to the nucleus of SMAD proteins 2 and 3 (Hu et al., 2018). As can be observed in Figures 6D–F, both SMAD 2 and SMAD 3 showed an increased translocation to the nucleus in the BLM treated groups if compared to sham group. Interestingly, BLM 30 U/kg treated mice showed also a statistically significant increase in the cytosolic fractions (Figures 6G–I), indicating not only an increased activation, but also a global increased expression of SMADs 2 and 3.

### Proinflammatory NLRP3 inflammasome pathway is affected in the lungs of mice with early ILD onset

Finally, we decided to investigate inflammation onset in the lungs of mice treated with BLM 30 U/kg. Inflammation, indeed, is known to have a pivotal role in the worsening of ILD (Behr et al., 2024), and among inflammatory pathways, NLRP3-inflammasome signaling is of particular interest in this field (Woo et al., 2023).

**FIGURE 7:**
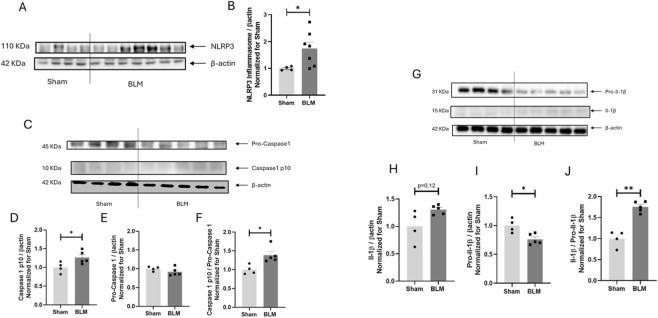
Inflammasome pathway activation in BLM mice. **(A)** Representative Western blot analysis of NLRP3-inflammasome expression in BLM and Sham mice lungs normalized to β-actin. **(B)** NLRP3-inflammasome expression in BLM mice normalized to β-actin and sham controls. **(C)** Representative Western blot analysis of Caspase 1 pro-caspase 10 expression in BLM and Sham mice lungs normalized to β-actin. **(D)** Caspase1 pro-caspase 10 expression in BLM mice normalized to β-actin and sham controls. **(E)** Pro-caspase 1 expression in BLM mice normalized to β-actin and sham controls. **(F)** Ratio of expression of Caspase 1/pro-caspase 10 in BLM mice normalized to pro-caspase 1 in BLM mice normalized to sham controls mice. **(G)** A representative Western blot analysis of Il-1 β expression in BLM and Sham mice lungs normalized to β-actin. **(H)** Il-1 β expression in BLM mice normalized to β-actin and sham controls. **(I)** Pro Il-1 β expression in BLM mice normalized to β-actin and sham controls. **(J)** Expression of Il-1 β normalized to pro Il-1 β in BLM mice normalized to Sham mice.

In our preclinical model of early ILD, we observed an overall activation of this pathway. Low dose of BLM, indeed, led to an overexpression of NLRP3 inflammasome (Figures 7A,B), to an increased expression of the active form of caspase 1 (Figures 7C–F) and finally to an overactivation of IL-1β (Figures 7G–J).

## Discussion

Bleomycin-induced lung fibrosis remains a cornerstone in preclinical ILD research due to its capacity to recapitulate key pathological features of human fibrotic lung diseases, including systemic sclerosis–associated ILD (SSc-ILD). Traditionally, disease severity in these models has been assessed through histopathological scoring and biochemical measurements of collagen; yet these approaches preclude longitudinal evaluation in individual animals. In this context, *in vivo* micro-computed tomography (micro-CT) has progressively emerged as a valuable adjunct to conventional histological and biochemical analyses, enabling longitudinal, non-invasive assessment of lung structural changes and therapeutic responses in the same animals. Micro-CT has been shown to correlate with quantitative histological measures such as Ashcroft score, collagen content, and alveolar air area in bleomycin-induced pulmonary fibrosis models, and to enable evaluation of disease progression over time, thereby reducing animal use and improving data quality in preclinical studies (Ravanetti et al., 2021; Ruscitti et al., 2017). As an example, in acute bleomycin intratracheal administration models, micro-CT has been shown to track disease progression and correlate imaging metrics with histology at early time points, such as 7, 14 and 21 days (Ruscitti et al., 2017).

A careful examination of the existing literature indicates that radiologically detectable changes in bleomycin-induced lung injury are most commonly reported at relatively high bleomycin doses or at advanced disease stages. This likely reflects the fact that micro-CT has mostly been used to detect clear, established changes in lung tissue, rather than to study the subtle early alterations that occur at lower bleomycin doses.

Consistent with this, several studies using systemic chronic bleomycin delivery via osmotic minipumps have relied on doses equal to or exceeding 60 U/kg to induce lung pathology that is readily detectable by conventional endpoints. Liang et al. described a progressive pattern of lung injury following continuous systemic bleomycin administration, with early inflammatory and epithelial changes evolving into parenchymal consolidation and architectural distortion at later stages of disease (Liang et al., 2015). Similarly, Watanabe et al. (2017) demonstrated a clear dose-dependent increase in pulmonary fibrosis, with significant collagen accumulation and subpleural fibrotic remodeling observed exclusively at high bleomycin doses (60–110 U/kg). In contrast, at lower doses (1–10 U/kg) no significant increases in lung hydroxyproline content or overt fibrotic architecture were detected, and only minimal tissue alterations were observed by histological analysis.

Nevertheless, even modest changes induced by lower bleomycin doses may be biologically meaningful, as they could correspond to the earliest phases of fibrotic remodeling that precede irreversible tissue reorganization. In this early therapeutic window, indeed, pharmacological intervention may effectively halt or reverse fibrotic progression before extensive extracellular matrix deposition and structural damage occur. In this framework, micro-CT holds considerable potential as a tool to capture these early events, provided that appropriate acquisition and analysis strategies are employed.

Ravanetti et al. extensively characterized a systemic bleomycin model delivered via osmotic minipumps and identified 60 U/kg as the optimal dose to reproducibly induce pulmonary and cutaneous fibrosis with predominant subpleural localization and macrophage-driven profibrotic remodeling (Ravanetti et al., 2020). Although lower doses (30 and 45 U/kg) were initially evaluated, subsequent analyses focused on the 60 U/kg regimen, highlighting the challenges associated with detecting robust fibrotic endpoints at sub-threshold doses using standard readouts. In a subsequent study (Ravanetti et al., 2021), the same group incorporated longitudinal micro-CT imaging to assess the antifibrotic efficacy of nintedanib, but again, a lung aeration impairment was detected only at high doses of bleomycin. Importantly, in this work the lung parenchyma was segmented into only two compartments—well-aerated and poorly aerated regions—an approach that is well suited for identifying advanced fibrotic remodeling but that might be less informative for capturing spatially heterogeneous or early-stage alterations.

There is, thereby, a compelling need for a refined preclinical model that better recapitulates the early stages of disease and allows pharmacological intervention within a clinically relevant therapeutic window. Building upon this well-established experimental framework, the present study introduces a refined imaging strategy specifically designed to investigate early fibrotic changes. We hypothesized that reducing the dose of BLM to 30 U/kg would return to a more gradual and physiologically relevant evolution of lung pathology. However, using conventional micro-CT image analysis methods—which classify lung parenchyma into only two regions (well-aerated vs. poorly aerated) — we were unable to detect early and small alterations in aeration induced by this lower dose of BLM. This result is in line with the above-mentioned literature. Despite this limitation, a reduction in body weight was observed in mice treated with 30 U/kg BLM at day 21, indicating systemic impact and prompting the need for an alternative, more sensitive imaging analysis protocol.

To overcome these limitations, we refined an enhanced segmentation strategy for micro-CT imaging, dividing the lung parenchyma into ten distinct aeration-based regions (based on HU from micro-CT images) rather than a binary classification. By implementing a more granular lung segmentation we were able to detect small yet significant changes in lung aeration patterns induced by a low bleomycin dose (30 U/kg) delivered via subcutaneous osmotic minipumps. Importantly, this approach requires only minimal adjustments to existing imaging workflows, facilitating its adoption in future preclinical studies.

To better contextualize the translational relevance of our imaging strategy, it is important to emphasize that our 10-segment micro-CT analysis is grounded in density-based quantitative CT principles that are widely applied in clinical ILD research (Iwasawa et al., 2023). Our method is based on Hounsfield Unit (HU)–derived attenuation values, which constitute a core component of clinical quantitative CT analysis used to measure subtle shifts in lung aeration distribution in early interstitial lung abnormalities. Conceptually, our approach parallels clinical density distribution analyses and regional quantitative CT methods used to capture early parenchymal remodeling.

To the best of our knowledge, only a few recent studies have explored refined analyses of lung parenchymal changes using micro-CT beyond conventional segmentation schemes in preclinical ILD models. For instance, Buccardi et al. developed a fully automated deep learning-based micro-CT approach in an acute bleomycin model, demonstrating improved precision in lung segmentation and longitudinal tracking of fibrosis progression (Buccardi et al., 2023). Similarly, in an aged murine model of bleomycin-induced fibrosis, longitudinal micro-CT was used to assess structural and functional biomarkers over time, highlighting age-related differences in fibrotic susceptibility and progression (Buseghin et al., 2024).

Although these works provide valuable insights into advanced imaging analyses, they differ in experimental design from the chronic systemic bleomycin exposure used in our study. Importantly, the imaging findings reported by Buccardi et al. are in line with our observations: the aeration curve described in their acute model shows a rightward shift (compared to control group) in parenchymal aeration metrics that closely overlaps with the shift observed in our chronic high-dose group (BLM+). This consistency suggests that refined imaging analyses can reveal subtle fibrosis-associated alterations across different bleomycin administration protocols, supporting the biological relevance of early parenchymal changes identified in our work.

A further advantage of this model is the temporal stability of the aeration profile. In mice treated with 30 U/kg BLM, impaired lung aeration remained stable for at least 7 days after the removal of BLM loaded osmotic pumps (between day 14 and day 21), defining a valuable window for pharmacological intervention/testing. This stability enhances the model’s translational relevance by allowing the assessment of therapeutic agents during a period that mirrors early disease onset, rather than in advanced fibrotic stages where therapeutic interventions are often less or not effective.

Moreover, molecular analyses conducted at day 21 revealed activation of both pro-fibrotic and pro-inflammatory pathways, confirming that low-dose BLM is sufficient to trigger representative key pathogenic mechanisms implicated in ILD. Pathophysiological steps in ILD include alveolar epithelial damage, fibroblast activation, and persistent fibrotic reaction. Differentiation of lung fibroblasts into myofibroblasts is a key step in the development of tissue fibrosis, and TGF-β is a key player in these processes, acting on both fibroblasts and alveolar epithelial cells (Saito et al., 2018). Myofibroblasts express α-smooth muscle actin (α-SMA) as a marker of activated fibroblasts, and are capable of extracellular matrix proteins production, including collagen, laminin, and fibronectin. The major cellular sources of TGF-β in pulmonary fibrosis are alveolar macrophages (differentiated into “pro-inflammatory” classic M1 macrophages or “profibrotic” M2a macrophages in ILD) (Kishore and Petrek, 2021) and T cells (Celada et al., 2018). The overexpression of growth factors like TGF-β leads to the activation of the downstream intracellular signal transduction pathways, such as the Smad-dependent signaling, closely related to the occurrence and development of ILD. The Smad-dependent signaling pathway implies that after TGF-β induction, the transduction protein Smad2 binds to Smad3, and subsequently phosphorylates and activates to form a trimer with Smad4 which transmits signals to the nucleus (Feng and Derynck, 2005).

In our study, the TGF-β/Smad signaling cascade was found to be upregulated, with increased nuclear translocation of Smad2 and Smad3. This pathway plays a central role in promoting fibroblast activation, epithelial-to-mesenchymal transition (EMT), and extracellular matrix (ECM) deposition, all of which are hallmarks of progressive pulmonary fibrosis (Meng et al., 2016; Dong Xu et al., 2003; Kolosova et al., 2010).

Concomitantly, we observed evidence of innate immune activation through upregulation of the NLRP3 (pyrin containing nucleotide-binding oligomerization domain) like receptor 3 inflammasome.

NLRP3 is highly expressed in leukocytes and upregulated in sentinel cells in response to pathogens or danger signals; NLRP3 inflammasome is the most deeply analyzed for its involvement in the innate and adaptive immune system as well as its contribution to several autoinflammatory and autoimmune diseases (Napodano et al., 2023). Among the various mechanisms involved, the purinergic receptor (P2X7R) appears to play a potentially significant role in NLRP3 activation (Kasper and Barth, 2017). P2X7R, which is expressed in damaged alveolar type I cells following tissue injury or death, may facilitate the formation of large non-selective pores in cells bearing P2X7R in their membrane. The opening of these pores could trigger the assembly and activation of the inflammasome complex (Napodano et al., 2023), though the precise mechanistic contribution of P2X7R to this pathway warrants further experimental investigation.

NLRP3 multiprotein complex, once assembled in response to cellular stress, mediates the cleavage of pro-caspase-1 into its active form, caspase-1, which in turn processes pro-IL-1β into mature IL-1β. This cytokine is a potent amplifier of inflammation and fibrosis, promoting leukocyte recruitment, fibroblast proliferation, and TGF-β expression, thereby contributing to an early self-sustaining fibrotic loop (Gasse et al., 2007; Riteau et al., 2012; Kim et al., 2014).

These findings underscore the translational relevance of our model, as it allows for the detailed dissection of early molecular events that bridge epithelial injury, immune signaling, and fibrogenesis. Notably, the engagement of both the TGF-β/Smad axis and the NLRP3/caspase-1/IL-1β pathway within the same temporal window highlights the convergence of fibrotic and inflammatory processes, which could be strategically targeted by combination therapies in future preclinical investigations.

Some limitations should be acknowledged. The present study provides longitudinal radiological and molecular characterization of early lung injury but does not include direct measurements of pulmonary mechanics (e.g., lung compliance). Gold-standard assessment of lung function in small animals, however, typically requires invasive procedures such as tracheostomy or orotracheal intubation combined with computer-controlled ventilation systems (Bonnardel et al., 2019). These approaches would have compromised the study by necessitating terminal procedures at intermediate time points. Future studies integrating refined micro-CT analysis with functional respiratory measurements would provide complementary validation of early-stage disease detection. Moreover, although refined micro-CT detected aeration redistribution at day 14, imaging alone cannot distinguish between inflammation, edema, or early fibrogenic remodeling. According to our experimental *in vivo* protocol, molecular validation was performed at day 21, but the absence of intermediate *ex vivo* analyses limits definitive biological interpretation of the early imaging changes. Future studies including day-14 validation would further clarify the temporal dynamics of these processes. Finally, the present study was conducted exclusively in female C57BL/6J mice; therefore, validation of this imaging approach across different strains and sexes would further strengthen its generalizability.

In summary, our study presents a novel *in vivo* protocol for the investigation of ILD, characterized by a reduced BLM dose and a refined micro-CT analysis pipeline. This model might successfully balance the need for pathological relevance with the capacity for early-stage therapeutic evaluation, positioning it as a promising tool for both mechanistic and translational research in ILD.

## Data Availability

The original contributions presented in the study are included in the article/supplementary material, further inquiries can be directed to the corresponding author.
